# Ambient Temperature Cycles Affect Daily Torpor and Hibernation Patterns in Malagasy Tenrecs

**DOI:** 10.3389/fphys.2020.00522

**Published:** 2020-05-28

**Authors:** Kathrin H. Dausmann, Danielle L. Levesque, Jens Wein, Julia Nowack

**Affiliations:** ^1^Functional Ecology, Institute of Zoology, University of Hamburg, Hamburg, Germany; ^2^School of Biology and Ecology, University of Maine, Orono, ME, United States; ^3^School of Biological and Environmental Sciences, Liverpool John Moores University, Liverpool, United Kingdom

**Keywords:** hibernation, torpor, body temperature, *zeitgeber*, tropics, *Echinops telfairi*

## Abstract

Hibernation and daily torpor (heterothermy) allow endotherms to cope with demanding environmental conditions. The depth and duration of torpor bouts vary considerably between tropical and temperate climates, and tropical hibernators manage to cope with a wider spectrum of ambient temperature (*T*_a_) regimes during heterothermy. As cycles in *T*_a_ can have profound effects on activity and torpor patterns as well as energy expenditure, we examined how these characteristics are affected by daily fluctuating versus constant *T*_a_ in a tropical hibernator, the lesser hedgehog tenrec (*Echinops telfairi*). Throughout the study, regardless of season, the tenrecs became torpid every day. In summer, *E. telfairi* used daily fluctuations in *T*_a_ to passively rewarm from daily torpor, which led to synchrony in the activity phases and torpor bouts between individuals and generally decreased energy expenditure. In contrast, animals housed at constant *T*_a_ showed considerable variation in timing and they had to invest more energy through endogenous heat production. During the hibernation season (winter) *E. telfairi* hibernated for several months in constant, as well as in fluctuating *T*_a_ and, as in summer, under fluctuating *T*_a_ arousals were much more uniform and showed less variation in timing compared to constant temperature regimes. The timing of torpor is not only important for its effective use, but synchronization of activity patterns could also be essential for social interactions, and successful foraging bouts. Our results highlight that *T*_a_ cycles can be an effective *zeitgeber* for activity and thermoregulatory rhythms throughout the year and that consideration should be given to the choice of temperature regime when studying heterothermy under laboratory conditions.

## Introduction

Limited food and water supply and unfavorable climatic conditions often require mammals to find means to reduce their dependency on energy and water availability. This holds particularly true for small animals in harsh climates as their small surface area to volume ratios lead to greater heat transfer and water loss. Hibernation, prolonged and daily torpor (combined here as heterothermy) are physiological strategies that allow endotherms to cope with such demanding conditions (Lyman et al., 1982). These physiological states are characterized by an active depression of metabolic rate (MR) and a change in set point of body temperature (*T*_b_, Geiser, 2004; Heldmaier et al., 2004), providing high savings in energy and water and a lowered *T*_b_ that can be close to ambient temperature (*T*_a_) during deep torpor (Ruf and Geiser, 2015). Daily torpor is limited to short bouts of less than 24 h, prolonged torpor bouts last for a few days, whereas hibernation consists of a series of approximately 1–2 week-long torpor bouts interspersed by active arousals, usually totaling several months (Geiser, 2004; Heldmaier et al., 2004; Nowack et al., 2020). Although heterothermy is best known, and probably more common in arctic and temperate climates, it also occurs in the tropics (Cossins and Barnes, 1996; Ruf and Geiser, 2015; Nowack et al., 2020). With increasing numbers of ecophysiological field studies there is more and more evidence of heterothermy in tropical mammals: e.g., bats (Stawski and Geiser, 2011; Reher et al., 2018), cheirogaleid lemurs (Dausmann, 2014), the bushbaby *Galago moholi* (Nowack et al., 2010), loris (Ruf et al., 2015; Streicher et al., 2017), tenrecs (Lovegrove and Génin, 2008; Oelkrug et al., 2013; Levesque et al., 2014), birds (McKechnie and Mzilikazi, 2011), echidnas, and marsupials (Grigg and Beard, 2000; Geiser and Körtner, 2010; Körtner et al., 2010). In fact, there are more mammalian orders with heterotherms than without, and it is likely that the capacity for heterothermy is the ancestral state in mammals (Grigg et al., 2004; Lovegrove, 2012a,b).

In contrast to arctic or temperate regions, the ultimate triggers of heterothermy in the tropics might not be as straight forward, and are more multifaceted than low temperature and low availability of food (see Geiser and Brigham, 2012; Nowack et al., 2017, 2020). The costs of endothermy might be less pronounced in many parts of the tropics, however, food and water can be scarce during all or some periods of the year. Thus, the water saving potential of hibernation and daily torpor becomes more important in the tropics (Macmillen, 1965; Cryan and Wolf, 2003; Schmid and Speakman, 2009). Reductions in MR, *T*_b_, food requirements and activity are accompanied by reductions in evaporative, fecal and urinary water loss, leading to substantial water savings (Cooper et al., 2005; Withers et al., 2012). Similarly, the time course and pattern of *T*_b_ during hibernation varies considerably between tropical and temperate climates. Arctic and temperate hibernators encounter very low *T*_a_ and subsequently exhibit very low *T*_b_ during hibernation, sometimes even below the freezing point (Barnes, 1989; Pretzlaff and Dausmann, 2012). Additionally, hibernacula of arctic and temperate hibernators are generally well insulated and temperature fluctuations are small within the hibernaculum (Arnold et al., 1991; Buck and Barnes, 1999). Tropical hibernators, on the other hand, use hibernacula with very variable degrees of insulation capacities, e.g., hollows in trees of varying heights and thicknesses, or underground sites at varying depths (Dausmann et al., 2004; Körtner et al., 2010; Blanco et al., 2013; Levesque et al., 2014; Lovegrove et al., 2014a). Therefore, depending on the choice of hibernaculum or resting site, i.e., well versus poorly insulated, tropical hibernators manage to cope with a wide spectrum of temperature regimes during hibernation: from constant to highly fluctuating temperatures, with elevated temperatures possibly enhanced by tropical solar radiation during the day. As *T*_b_ usually approximates *T*_a_ during hibernation, this flexibility is also reflected in *T*_b_ (Dausmann et al., 2004; Kobbe and Dausmann, 2009; Canale et al., 2012; Levesque et al., 2014; Reher et al., 2018). For example, the range of daily *T*_a_ fluctuations affects the hibernation pattern in the lemur *Cheirogaleus medius* (Dausmann et al., 2005) and many heterotherms use the daily *T*_a_ fluctuations to assist warming up from daily and prolonged torpor or hibernation bouts (Ortmann et al., 1997; Schmid, 2000; Mzilikazi et al., 2002; Turbill and Geiser, 2008; Warnecke et al., 2008; Kobbe and Dausmann, 2009; Warnecke and Geiser, 2010; Thompson et al., 2015). However, *T*_a_ cycles not only help rewarming, they also act as a *zeitgeber*, influencing activity patterns (Pohl, 1998; Vivanco et al., 2010).

We therefore sought to characterize the effects of differing *T*_a_ patterns on the thermophysiology of a tropical hibernator, the lesser hedgehog tenrec (*Echinops telfairi*). We aimed to evaluate how the choice of hibernaculum (i.e., insulation capacity) influences hibernation parameters in the wild by examining how daily fluctuating *T*_a_ versus constant *T*_a_ affects patterns of daily torpor and hibernation and energy expenditure. As the previous measures of cost of hibernation under constant *T*_a_ conditions may have overestimated the total frequency and cost of rewarming in tropical hibernators, we measured metabolic rate to test if *T*_a_ fluctuations are used to assist with warming during arousals. Finally, by simulating a range of summer and winter temperatures, we aimed to analyse how hibernation patterns and energy expenditure are affected by variable and changing temperatures during hibernation.

## Materials and Methods

### Study Species

*Echinops telfairi* (Martin, 1838) is a small (135 g) nocturnal insectivorous member of the family Tenrecidae and endemic to Madagascar (Eisenberg and Gould, 1969). It uses daily torpor during the austral summer, and hibernates during the winter. It has one of the lowest reported euthermic *T*_b_ of any eutherian mammal and is highly thermally labile (Scholl, 1974; Clarke and Rothery, 2008; Lovegrove and Génin, 2008). In Madagascar, *E. telfairi* rests and hibernates in tree hollows, dead trees or under leaf litter (Eisenberg and Gould, 1969; Soarimalala and Goodman, 2011). Neither offer a particularly well-insulated resting site and it can thus be assumed that they experience fluctuating *T*_a_ year-round.

Eighteen female and nine male adult, laboratory-bred lesser Malagasy hedgehog-tenrecs (*E. telfairi*; 3–5 years old during the experiments) were used for the experiments over a two year time period. The animals were acquired from the Ludwig-Maximilians-University Munich, where they had been bred for over 30 years and fully acclimated to northern hemisphere seasonal rhythms (e.g., Künzle, 1998). All animals were earmarked or marked with an injectable micro transponder (ID-100, Trovan, Usling GmbH, Weilerswist, Germany), to unambiguously identify individuals.

### Experimental Setup

The animals were kept in separate cages (35 × 21 × 35 cm, L × W × H) in a climate chamber (Type TCR + 2, Weiss Technik, Reiskirchen, Germany) during the experiments and weighed regularly. The cages were equipped with wooden nest boxes (14 × 20 × 14 cm), wood chips, a hamster wheel and other environmental enrichment. Food (mealworms, cockroaches, wet canned cat food, dry dog food, dry hedgehog food, boiled egg and fresh fruit) and water were provided *ad libitum*. To test for the effects of *T*_a_ on torpor patterns the animals were exposed to five different temperature treatments, two during the animals’ summer and three in their winter. Temperatures were chosen to match actual climatic conditions of *E. telfairi* in their natural resting sites (Jury, 2003; Dausmann and Blanco, 2016). During summer, day length (simulated by ambient lighting) and humidity were adjusted to 13 h and 70%, respectively. *T*_a_ was either held constant at 24°C (S_const__24_) or fluctuating between 19°C during the dark phase and 28°C during the light phase (S_fluc__19__–__28_). In winter day length was reduced to 11 h and humidity to 40%, constant *T*_a_ was set at 18 or 12°C (W_const__18_ and W_c__onst__12_), and fluctuating *T*_a_ varied from 14°C during the dark phase to 24°C during the light phase (W_fluc__14__–__24_). The constant *T*_a_s 24 and 18°C were chosen as the middle between the minimum and maximum of the fluctuating *T*_a_ of the respective season (as would be found in a very well insulated resting site). Additionally, a constant *T*_a_ of 12°C was included during winter to investigate responses and limitations of *E. telfairi* to a constant temperature regime at the lower end of temperatures in their resting sites. Animals were randomly assigned to the different experimental treatments and were used in multiple experiments. Each treatment lasted for a minimum of two weeks and the sequence of experimental treatments within each season was randomized.

### Measurement of Ambient and Skin Temperature

Skin temperature (*T*_skin_) and *T*_a_ were measured with temperature data loggers (3.3 g; iButton, DS1922L, Maxim Integrated Products, Inc., Sunnyvale, United States) set to logging intervals of 15 min and a resolution of 0.0625°C. The data loggers were taped to the shaved animals’ abdominal regions with medical tape (Fixomull stretch, BSN medical, Hamburg, Germany), which did not restrict the tenrecs’ movements in any way and remained in close contact to the skin during activity. External temperature loggers give reliable approximation of *T*_b_, especially during resting and torpor phases, when the animals are curled up with the logger positioned inside (Barclay et al., 1996; Dausmann, 2005). When loggers fell off (mainly during activity phases), they were re-taped to the animals without any apparent disturbance before they became torpid again. As the tenrecs (and cages) were checked daily, we found detached loggers within 24 h and the corresponding data were omitted from analyses.

**FIGURE 1:**
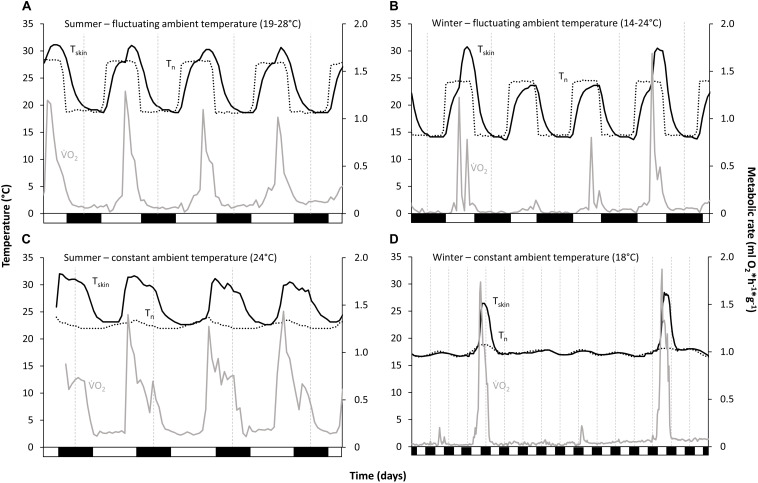
Torpor and arousal/activity phase pattern of *Echinops telfairi* for four experimental treatments. **(A,B)** fluctuating temperature conditions (summer and winter), **(C,D)** constant temperature conditions (summer and winter). Tenrecs became torpid every day; under fluctuating temperature conditions, they used these fluctuations for passively warming from torpor until maximum nest box temperature, whereas under constant temperature conditions they had to rely on endogenous heat production. Black line: skin temperature; dotted line: nest box temperature; gray line: metabolic rate; vertical dashed lines indicate midnight, black boxes on the *x*-axis the scotophase.

Loggers for recording *T*_a_ were fixed to the inside of each cage (to control the preciseness of the climate chamber) and each nest box (*T*_n_ in the analyses). Temperature readings were averaged for every hour. As T_skin_ of the tenrecs is very flexible and sometimes low even in the non-torpid state, it was not possible to define a torpor/non-torpor threshold for *T*_skin_. However, *T*_skin_ was always either almost at *T*_a_, or distinctly above it. Thus, the animals were considered to be torpid when *T*_skin_ was at or only slightly above *T*_n_ (*T*_skin_–*T*_n_ ≤ 2°C), as confirmed by the obvious drop and increase in MR at the beginning and end of each torpor bout and of each activity phase (summer) or arousal (winter) (see Figure 1). The term “arousals” in this study thus includes the (active) rewarming phase as well as periods of activity with normothermic *T*_skin_ (especially during summer) and parts of the cooling phase.

### Measurement of Metabolic Rate

Metabolic rate was measured via oxygen consumption with two differential oxygen analyzers (OxBox, FIWI, Vienna University, Vienna, Austria). Air was drawn at a rate of 50 L/h from the nest boxes, which served as respiratory chambers and were connected to the oxygen analyzers with airtight tubes (Tygon tubing ST, Saint-Gobain, Charny, France). Dividers in the middle of the nest boxes lessened air mixing between ambient air and the inside of the nest boxes. A gas splitter (RM Gas Flow Multiplexer, Sable Systems, Las Vegas, NV, United States) was used to rotate air flow amongst four different nest boxes. Thus, a total of eight animals could be measured at the same time using the two oxygen analyzers. During a 1-h long rotation cycle, all nest boxes were measured for 15 min and data stored every minute, and once per hour reference air was measured for 5 min to obtain a baseline value. Depending on the start of the measurement, the reference air measurement fell into the measurement period of different animals. Air leaving the nest boxes was dried with silica gel (Silica Gel Orange, 2–5 mm, Carl Roth, Karlsruhe, Germany) before entering the analyzer and the flow meter. The oxygen analyzers were calibrated with calibration gas mixtures produced with a gas-mixing pump (2KM300/a, H. Wösthoff GmbH, Bochum, Germany) and values were corrected for the CO_2_ effect with: ΔVol%O_2_ = ΔO2 + 0.0818^∗^ΔO_2_. Oxygen consumption was calculated using the following equation: $\dot{\text{V}}\,{\text{O}}_{2}$ [ml O_2_^∗^h^–1^] = ΔVol%O_2_^∗^ flow [L^∗^h^–1^] ^∗^ 10 (Heldmaier and Steinlechner, 1981) and was converted to $\dot{\text{V}}\,{\text{O}}_{2}$ per gram body mass (ml O_2_^∗^g^–1*^h^–1^). Mass used in the analysis was calculated as the average of the body mass before and after the experimental periods. Mean hourly values of oxygen consumption were calculated and synchronized with corresponding temperature readings. For each daily torpor bout, hibernation bout, arousal and activity phase minimum, maximum and average oxygen consumption and *T*_skin_ were calculated. Additionally, the average oxygen consumption for the warming phase of each torpor bout was calculated. Oxygen consumption could only be measured as torpid or resting metabolic rate (RMR) in most cases, as measurements were only possible when the animals were in the nest boxes and therefore total energy budgets, including activity could not be calculated. Due to technical problems, $\dot{\text{V}}\,{\text{O}}_{2}$ is not available for W_const__12_.

### Statistical Methods

If not stated otherwise, values are given as mean ± standard deviation, with *N* denoting the number of individuals tested per treatment, *n* the number of observations. As the aim of our study was to identify responses on a population level and due to the uneven distribution of sexes within and across treatments, the data from both sexes were pooled. All statistical procedures were done using R (R Development Core Team, 2018). Differences in weight were tested with a *t*-test after the data were checked with a Kolmogorov–Smirnov test for normal distribution. To account for an unbalanced data set, differences in minimum $\dot{\text{V}}\,{\text{O}}_{2}$ and *T*_skin_ during torpor, torpor bout duration (TBD), maximum $\dot{\text{V}}\,{\text{O}}_{2}$, average $\dot{\text{V}}\,{\text{O}}_{2}$ and *T*_skin_ during arousal and length of the activity period were tested via generalized estimation equations with a Gaussian error structure and an autoregressive, AR1, correlation structure (“geeglm” in library “geepack,” Yan, 2002; Yan and Fine, 2004; Halekoh et al., 2006), followed by type 1 ANOVA. Individual ID was included as a random effect to adjust for repeated measurements. To test for statistical differences of $\dot{\text{V}}\,{\text{O}}_{2}$, we used total $\dot{\text{V}}\,{\text{O}}_{2}$ per animal as the response variable and adjusted for body mass by including it as a covariate. Mass-specific metabolic rates are given for descriptive purposes but were not used in statistical analyses. *Post hoc* analyses were performed as Tukey tests (“glht” in library “multcomp,” Hothorn et al., 2008).

Rayleigh tests were used to determine whether circular data (timing of arousals) differed significantly from random (“rayleigh.test” in library “circular,” Jammalamadaka and Sengupta, 2001). Watson two-tailed tests were performed to test differences between the timing of arousals between treatments (“watson.two.test” in “circular”). To examine differences in the variance of arousal timing, we performed generalized estimation equations as described above. Variance was calculated as the individual deviation from the mean time of arousal start (minutes from midnight) per treatment. Resultant probability values were compared to an α-value of 0.05.

## Results

### Behavior and Body Mass

Corresponding with their nocturnal lifestyle, all animals were active during the late day and early night throughout summer and used short bouts of torpor during the rest phase on a daily basis. Only one female animal showed an activity phase exceeding 24 h on two occasions during S_const__24_ (28 and 40 h long). During winter, all *E. telfairi* hibernated for several months (*N* = 27) and spent most time inside their nest boxes, even during arousals, although some individuals briefly left their boxes to drink. Most animals did not eat during winter (see below). Average body mass during summer was 156 ± 28 g (*N* = 27). Mean body mass fell to 126 ± 24 g (*N* = 23) during the winter (*t*-test, *t* = 10.616, *p* < 0.001).

In a separate study, food intake of the study individuals across the seasons was measured. During winter, most tenrecs did not eat. If they ate, it was only a small fraction of the amount they ate during summer (about 2.7 g mealworms and 1.4 g banana (dry weights) per month in summer vs. 0.2 g mealworms and 0.3 g banana per month in winter (*N* = 6, *n* = 18; *t*-test, *t* = −26.393, *p* < 0.001 for mealworms; *t*-test, *t* = −2.696, *p* = 0.043 for banana, Lund, 2009).

### Torpor Duration, Minimum *T*_skin_, and Oxygen Consumption

Torpor bout length (TBD), minimum oxygen consumption ($\dot{\text{V}}\,{\text{O}}_{2}$), and minimum *T*_skin_ were significantly different between the five experimental conditions (for data and statistical analyses see Tables 1, 2). During both summer temperature treatments *E. telfairi* entered short bouts of torpor every day during the first part of the resting phase (Figures 1A,C). Minimum *T*_skin_ closely resembled minimum *T*_n_ under both summer conditions (S_const__24_ and S_fluc__19__–__28_) and hourly mean minimum *T*_skin_ thus was significantly lower during S_fluc__19__–__28_ than during S_const__24_ (Tables 1, 2). Furthermore, TBD was almost double under the fluctuating condition and thus significantly longer than during S_const__24_ (Tables 1, 2).

**TABLE 1:** Torpor and arousal/activity phase characteristics of *Echinops telfairi* for all five experimental treatments.

		**Hibernation/daily torpor bouts**			**Arousals/activity phases**				
**Treatment**		**Duration (h)**	***T*_skin_ min (°C)**	**$\dot{\text{V}}\,{\text{O}}_{2}$ min (ml O_2_ g^–1^h^–1^)**	**Duration (h)**	***T*_skin_ max (°C)**	**$\dot{\text{V}}\,{\text{O}}_{2}$ average (ml O_2_ g^–1^h^–1^)**	**$\dot{\text{V}}\,{\text{O}}_{2}$ max (ml O_2_ g^–1^h^–1^)**	**Rewarming $\dot{\text{V}}\,{\text{O}}_{2}$ (ml O_2_ g^–1^h^–1^)**
S_flucl__9__–__28_	Mean	15.3 ± 0.9^a^	18.8 ± 0.3^a^	0.03 ± 0.03^a^	6.7 ± 1.0^a^	29.8 ± 1.0^a^	0.36 ± 0.12^a^	0.95 ± 0.28^a^	0.45 ± 0.18^a^
	Range	11–25	17.7–19.7	0.01–0.14	4–8	27.9–31.7	0.1–0.59	0.21–1.69	0.08–0.87
	*N*, *n*	*N* = 8, *n* = 36		*N* = 8, *n* = 32	*N* = 8, *n* = 37		*N* = 8, *n*-33		*N* = 8, *n*-33

S_const__24_	Mean	8.5 ± 2.1^b^	23.0 ± 0.6^b^	0.14 ± 0.04^b^	14.5 ± 9.9^b^	31.2 ± 0.7^b^	0.62 ± 0.19^b^	1.38 ± 0.63^a,b,c^	0.93 ± 0.49^b,c^
	Range	1–14	21.7–25.4	0.01–0.26	4–40	29.1–32.8	0.3–1.05	0.42–3.43	0.14–1.68
	*N*, *n*	*N* = 6, *n* = 28		*N* = 6, *n* = 25	*N* = 6, *n* = 28		*N* = 6, *n* = 25		*N* = 6, *n* = 13

W_fluc__14__–__24_	Mean	88.1 ± 117.7^c^	13.3 ± 0.8^c^	0.03 ± 0.01^a^	11.8 ± 4.0^c^	29.4 ± 1.1^a^	0.26 ± 0.11^c^	0.89 ± 0.55^a,c^	0.48 ± 0.27^a,c^
	Range	6–359	11.0–14.2	0.01–0.05	4–12	24.7–31.4	0.07–0.63	0.11–3.09	0.07–0.82
	*N*, *n*	*N* = 13, *n* = 43		*N* = 13, *n* = 41	*N* = 10, *n* = 40		*N* = 10, *n* = 39		*N* = 10, *n* = 39

W_const__18_	Mean	100.0 ± 51.6^ce^	17.0 ± 0.4^d^	0.09 ± 0.07^c^	9.4 ± 2.3^c^	28.0 ± 1.1^c^	0.68 ± 0.26^b^	1.50 ± 0.64^b^	1.00 ± 0.41^b^
	Range	16–194	15.9–18.1	0.01–0.38	3–17	23.5–30.1	0.06–1.12	0.15–2.74	0.43–1.76
	*N*, *n*	*N* = 18, *n* = 44		*N* = 17, *n* = 29	*N* = 15, *n* = 39		*N* = 12, *n* = 19		*N* = 7, *n* = 18

W_const__12_	Mean	204.7 ± 75.3^d^	11.3 ± 0.3^e^	n/a	12 ± 7.1^†^	26.6 ± 0.1^†^	n/a	n/a	n/a
	Range	83–287	10.7–12.2	n/a	7–17	26.5–26.7	n/a	n/a	n/a
	*N*, *n*	*N* = 8, *n* = 12		n/a	*N* = 2, *n* = 2		n/a	n/a	n/a

*Values are given as mean ± SD. *N* denotes the number of individuals tested per treatment, *n* the number of observations. Differing letters indicate statistical differences. †Not enough data for statistical analysis. S_*fluc19–28*_: summer, fluctuating condition (19–28°C); S_*const24*_: summer, constant condition (24°C); W_*fluc14–24*_: winter, fluctuating condition (14–24°C); W_*const18*_: winter, constant condition (18°C); W_*const14*_: winter, constant condition (14°C); n/a: not applicable.*

**TABLE 2:** Statistical parameters for ANOVA and Tukey *post hoc* tests for torpor and arousal/activity phase characteristics of *Echinops telfairi* for all five experimental treatments.

		**Hibernation/daily torpor bouts**			**Arousals/activity phases**				
**Treatment**		**Duration (h)**	***T*_skin_ min (°C)**	**$\dot{\text{V}}$O_2_ min (ml O_2_ g^–1^h^–1^)**	**Duration (h)**	***T*_skin_ max (°C)**	**$\dot{\text{V}}$O_2_ average (ml O_2_ g^–1^h^–1^)**	**$\dot{\text{V}}$O_2_ max (ml O_2_ g^–1^h^–1^)**	**Rewarming $\dot{\text{V}}$O_2_ (ml O_2_ g^–1^h^–1^)**
ANOVA	df	4	4	3	3	3	3	3	3
	χ^2^	183	5007	85.9	54.9	88.8	43.1	8.05	18.09
	*p*	<0.001	<0.001	<0.001	<0.001	<0.001	<0.001	0.045	<0.001

S_fluc19–28_ vs. S_const24_	*z*	5.45	20.6	5.25	4.01	3.89	3.4	1.89	2.58
	*p*	<0.001	<0.001	0.001	<0.001	<0.001	0.004	0.225	0.045

S_fluc19–28_ vs. W_fluc14–24_	*z*	2.74	41.4	1.28	4.20	1.16	2.75	0.19	0.94
	*p*	0.0388	<0.001	0.553	<0.001	0.649	0.028	0.100	0.774

S_fluc19–28_ vs. W_const18_	*z*	9.87	17.0	2.90	5.66	5.51	4.21	3.11	4.35
	*p*	<0.001	<0.001	0.018	<0.001	<0.001	<0.001	0.010	<0.001

S_fluc19–28_ vs. W_const12_	*z*	7.77	54.5	n/a	n/a	n/a	n/a	n/a	n/a
	*p*	<0.001	<0.001						

S_const24_ vs. W_fluc14–24_	*z*	3.21	42.0	8.06	3.27	4.93	5.36	1.67	2.20
	*p*	0.0078	<0.001	<0.001	0.005	<0.001	<0.001	0.333	0.116

S_const24_ vs. W_const18_	*z*	10.65	28.2	2.57	2.65	9.33	1.22	0.95	0.60
	*p*	<0.001	<0.001	0.046	0.032	<0.001	0.603	0.775	0.928

S_const24_ vs. W_const12_	*z*	7.79	50.1	n/a	n/a	n/a	n/a	n/a	n/a
	*p*	<0.001	<0.001						

W_fluc14–24_ vs. W_const18_	*z*	2.27	23.2	4.64	2.32	4.52	5.95	2.79	3.80
	*p*	0.1159	<0.001	<0.001	0.077	<0.001	<0.001	0.0259	<0.001

W_fluc14–24_ vs. W_const12_	*z*	5.35	12.5	n/a	n/a	3.85	n/a	n/a	n/a
	*p*	<0.001	<0.001			<0.001			

W_const18_ vs. W_const12_	*z*	4.35	38.9	n/a	n/a	3.23	n/a	n/a	n/a
	*p*	<0.001	<0.001			0.0087			

*S_fluc19–28_: summer, fluctuating condition (19–28°C); S_const24_: summer, constant condition (24°C); W_fluc14–24_: winter, fluctuating condition (14–24°C); W_const18_: winter, constant condition (18°C); W_const14_: winter, constant condition (14°C); n/a: not applicable.*

Animals hibernated in all winter conditions. The general hibernation pattern was similar during the W_const__12_ and W_const__18_ treatments. Animals entered torpor bouts of varying lengths during which *T*_skin_ was fairly constant and close to *T*_n_, alternating with periodic arousals (Figure 1B). Torpor bouts during winter were significantly longer than during both summer conditions (Tables 1, 2). Some torpor bouts were interrupted by the end of the experiment and therefore could have even been longer. Torpor bouts lasted twice as long and significantly longer in W_const__12_ as in W_const__18_ (Tables 1, 2). In fluctuating winter conditions, *T*_skin_ closely tracked the *T*_n_ cycle during hibernation bouts passively, with the lowest *T*_skin_ recorded as 10.7°C under W_const__12_ (Figure 1D and Table 1; climate chambers did sometimes deviate a little bit from the set temperature, in this case *T*_a_ was slightly cooler than 12°C). Arousals were more frequent in fluctuating than in constant winter temperatures and the hibernation bouts were highly variable in length (range: 6–359 h) and significantly different to the other treatments (except W_const__18_; Tables 1, 2). The differences in hourly mean minimum *T*_skin_ were significantly different for all five experimental groups (Tables 1, 2).

Minimum $\dot{\text{V}}\,{\text{O}}_{2}$ during torpor was significantly related to the minimum experimental temperature, i.e., lowest at W_fluc__14__–__24_ and S_fluc__19__–__28_ (0.03 ml O_2_^∗^g^–1*^h^–1^ for both) and more than four-fold higher under S_const__24_ (0.14 ml O_2_^∗^g^–1*^h^–1^; Tables 1, 2; Figure 1). Minimum $\dot{\text{V}}\,{\text{O}}_{2}$ differed significantly between all treatments except between both fluctuating conditions (Tables 1, 2).

### Arousals, Maximum *T*_skin_, and Oxygen Consumption

Animals used daily *T*_n_ fluctuation for passively rewarming from torpor and only activated endogenous heating after *T*_skin_ reached the high daytime *T*_n_ passively (Figures 1A,B). At the end of a torpor bout (which coincided with the start of the active rewarming and the initiation of activity phase), $\dot{\text{V}}\,{\text{O}}_{2}$ increased sharply and remained high until the end of the activity phase when it quickly dropped again (Figure 1). Maximum $\dot{\text{V}}\,{\text{O}}_{2}$ during arousals or activity phases, average $\dot{\text{V}}\,{\text{O}}_{2}$ and maximum *T*_skin_ reached during arousals and the length of the arousal/activity phase differed significantly between the temperature conditions (Tables 1, 2). Maximum $\dot{\text{V}}\,{\text{O}}_{2}$ and average $\dot{\text{V}}\,{\text{O}}_{2}$ during arousals were highest for S_const__24_ and W_const__18_ ($\dot{\text{V}}\,{\text{O}}_{2}$ max: 1.38 ml O_2_^∗^g^–1*^h^–1^ and 1.50 ml O_2_^∗^g^–1*^h^–1^; $\dot{\text{V}}\,{\text{O}}_{2}$ ave: 0.62 ml O_2_^∗^g^–1*^h^–1^ and 0.68 ml O_2_^∗^g^–1*^h^–1^; no significant differences between the two for either variable, Table 2), where all heating had to be endogenously initiated, and lowest during W_fluc__14__–__24_, where passive heating over most of the *T*_skin_ increase reduced energy expenditure (0.89 ml O_2_^∗^g^–1*^h^–1^), followed by S_fluc__19__–__28_ (0.95 ml O_2_^∗^g^–1*^h^–1^). While W_fluc__14__–__24_ and W_const__18_ were significantly different for maximum $\dot{\text{V}}\,{\text{O}}_{2}$, the difference between S_fluc__19__–__28_ and S_const__24_ was not significant (Table 2). All other treatments except S_const__24_ and W_const__18_ were significantly different from each other (Tables 1, 2). Thus, the high levels of maximum $\dot{\text{V}}\,{\text{O}}_{2}$ during active heating under constant temperature conditions carried over into average $\dot{\text{V}}\,{\text{O}}_{2}$ during arousals and activity phases.

$\dot{\text{V}}\,{\text{O}}_{2}$ during rewarming in the fluctuating temperature conditions (0.45 ml O_2_^∗^g^–1*^h^–1^ in summer and 0.48 ml O_2_^∗^g^–1*^h^–1^ in winter), including passive heating phases, was only about half, and significantly lower than that of the active heating phases observed under the constant conditions which relied exclusively on endogenous heat production (Tables 1, 2). Arousal rewarming $\dot{\text{V}}\,{\text{O}}_{2}$ during S_const__24_ and W_const__18_ was significantly higher than S_fluc__19__–__28_ and W_fluc__14__–__24_, respectively (Tables 1, 2) indicating energy saved by the use of passive heating. There were, however, no difference between the two fluctuating conditions (S_fluc__19__–__28_ and W_fluc__14__–__24_) and the two constant conditions (S_const__24_ and W_const__18_, Table 2).

**FIGURE 2:**
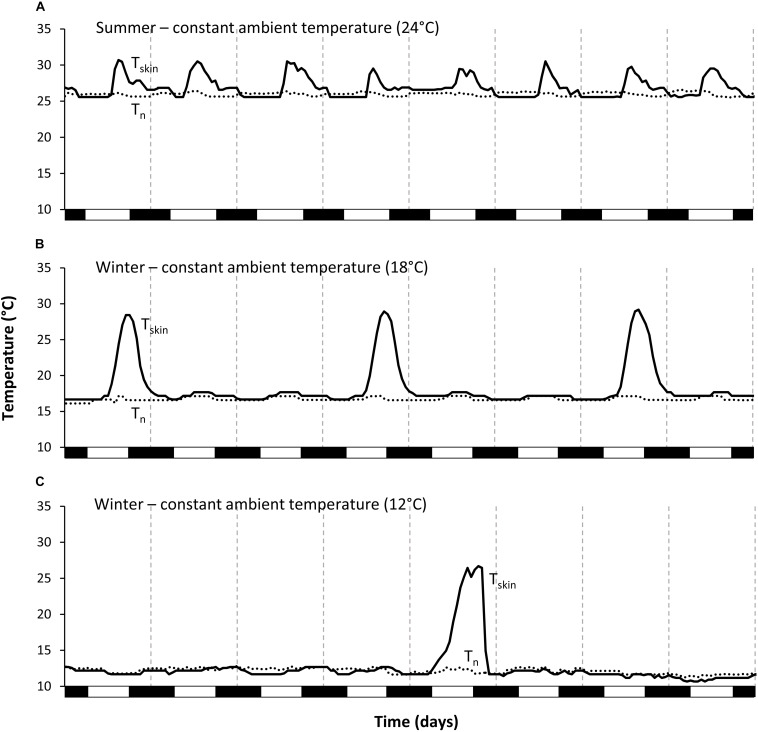
Examples of typical sequences of skin temperature of one individual *Echinops telfairi* during the different constant treatments over eight days. **(A)** Summer 24°C, **(B)** winter 18°C, and **(C)** winter 12°C. During summer, tenrecs aroused daily to normothermic skin temperature; during winter, torpor bouts became longer, even more so at the lower ambient temperature. Black line: skin temperature; dotted line: nest box temperature; vertical dashed lines indicate midnight, black boxes on the *x*-axis the scotophase.

*T*_skin_ followed the pattern of $\dot{\text{V}}\,{\text{O}}_{2}$ with a lag time that depended on the experimental condition (Figure 1). Hourly mean maximal *T*_skin_ during arousals was highest for S_const__24_ (Table 1); the highest overall recorded *T*_skin_ was 32.8°C. *T*_skin_ in the other treatments was slightly, but significantly lower (Tables 1, 2). In the S_const__24_ condition, the animals maintained elevated *T*_skin_ for about 14 h per day during the activity phase, but only for about 9 h, and significantly shorter, under S_fluc__19__–__28_, due to the passive heating phase under the fluctuating conditions (Table 2). Arousal or activity phases were significantly shortest in the S_fluc__19__–__28_ treatment (about 7 h; Table 2) and intermediate in the W_fluc__14__–__24_ treatment (Figure 2).

**FIGURE 3:**
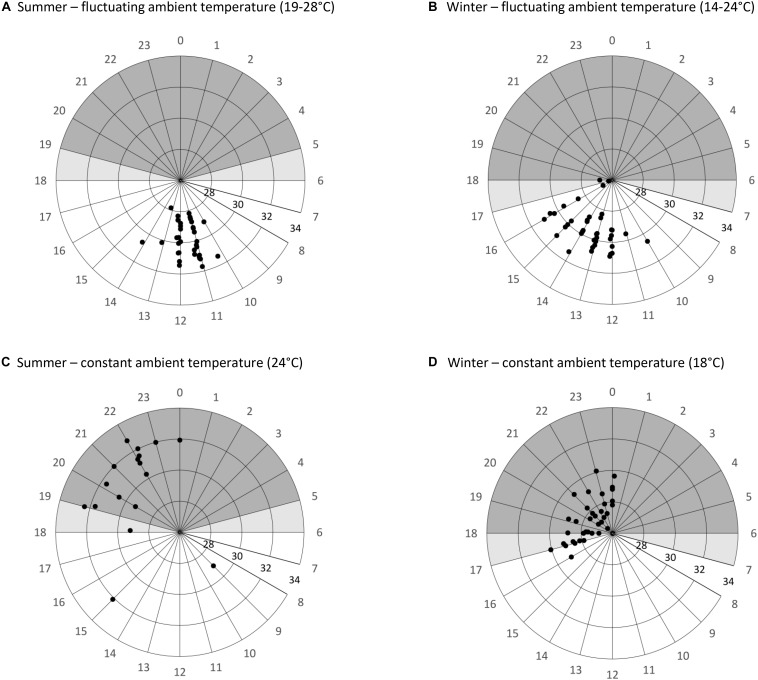
Timing of arousals (initiation of endogenous heat production) and maximum skin temperature during arousals/activity phases of *Echinops telfairi* for four experimental treatments **(A–D)**. Data shown indicate the time of day (degrees) and level of maximum skin temperature (°C; radial distance). Shaded areas illustrate the scotophase, lighter shaded areas the transitional times between night and day phase. Under fluctuating conditions **(A,B)**, arousals are more synchronous and initiated when *T*_skin_ passively reaches maximal *T*_n_ well before the beginning of the scotophase. Under constant conditions **(C,D)**, the start of arousals is scattered over a longer time period, but almost always only after the beginning of the scotophase. Maximal *T*_skin_ is highest during the activity phases under constant summer condition (24°C), and lowest and least variable during the arousals under constant winter conditions (18°C).

**FIGURE 4:**
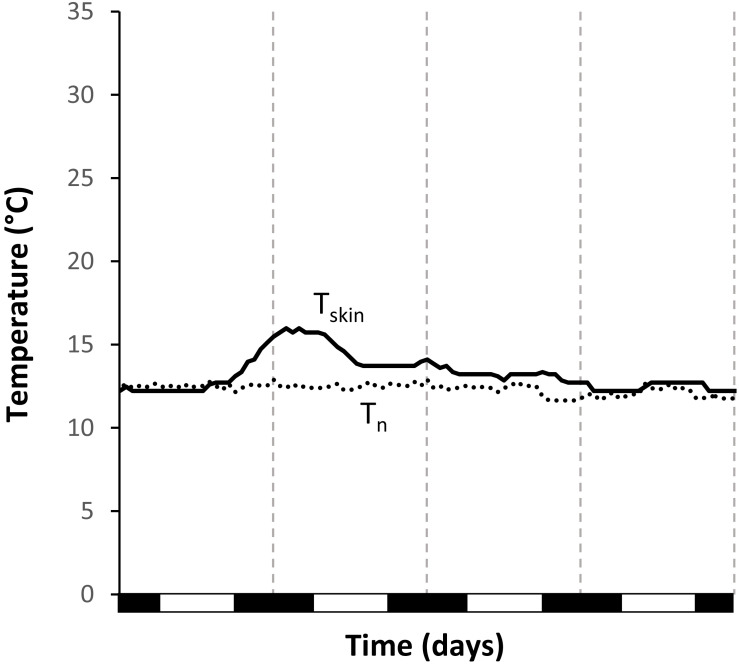
Unsuccessful attempt at rewarming from torpor of *Echinops telfairi* under constant temperature treatment in winter (W_const__12_). During this event, skin temperature reached a maximum of only 14.7°C and only slowly returned to the level of nest box temperature over two days. Black line: skin temperature; dotted line: nest box temperature; vertical dashed lines indicate midnight, black boxes on the x-axis the scotophase.

The timing of the arousals differed significantly from a random distribution for all treatments (Figure 3). We excluded W_cost__12_ from arousal data analysis as only two full arousals were recorded. However, there were three unsuccessful attempts at rewarming under this condition (Figure 4), which were never observed in any other treatment. In summer, animals under S_const__24_ started to rewarm at 18:43 ± 02:24 h (*N* = 6, *n* = 28; Rayleigh test: *r* = 0.7372, *p* < 0.001) and reached their maximum *T*_skin_ about 2 h later. Animals under S_fluc__19__–__28_ used daily *T*_n_ fluctuation for passively rewarming from torpor and only activated endogenous heating on average at 11:34 ± 00:35 h (*N* = 8, *n* = 37; Rayleigh test: *r* = 0.978, *p* < 0.001; Figure 3), which was significantly earlier than under the constant summer treatment (Watson’s test: *x* = 1.3308, *p* < 0.001; Figure 3).

During winter, arousals were less frequent, and even more so during the constant treatments. During W_const__12_ only two arousals were recorded and those varied considerably in timing (15:00 and 22:00 h). There was considerable variation in the timing of arousals under W_const__18_. Arousals for W_const__18_ started at 19:54 ± 04:27 h (*N* = 16, *n* = 40; Rayleigh test: *r* = 0.896, *p* < 0.001) and animals needed between four to 6 h to rewarm. For W_fluc__14__–__24_ arousals started at 13:51 ± 01:20 h, after *T*_skin_ had passively reached high daytime *T*_n_ (*N* = 10, *n* = 41; Rayleigh test, *r* = 0.896, *p* < 0.001; Figure 3). Arousals started more synchronously during both fluctuating treatments than under constant *T*_a_, i.e., the start time of the arousals was less variable (ANOVA, df = 3, χ^2^ = 51.3, *p* > 0.001; *z* > 2.82, *p* < 0.0212; Figure 3); while arousals were even more synchronized during S_fluc__24_ than W_fluc__18_ (deviation from mean 0.43 ± 0.35 min (*N* = 9, *n* = 38) and 1.06 ± 0.82 min (*N* = 10, *n* = 41), respectively; *z* = 4.08, *p* > 0.001), there were no significant differences between the two constant treatments (deviation from mean S_const__24_ 1.62 ± 0.79 min (*N* = 7, *n* = 29) and W_const__18_ 2.43 ± 1.72 min (*N* = 16, *n* = 40); z = 0.20, *p* = 0.9970). In general, in all analyzed parameters, $\dot{\text{V}}\,{\text{O}}_{2}$ was always higher in the constant than in the fluctuating conditions (Table 1).

## Discussion

Temperature regime was found to have a significant effect on hibernation patterns of the highly heterothermic *E. telfairi* highlighting the importance of incorporating realistic temperatures in the study of hibernation. The tenrecs entered torpor every day and fluctuating temperatures acted as a *zeitgeber* that allowed for more synchrony in activity between individuals as well as lower costs of arousal from torpor. Under fluctuating summer *T*_a_ (S_fluc__19__–__28_) conditions, *E. telfairi* was able to lower its $\dot{\text{V}}\,{\text{O}}_{2}$ during daily resting phases to the same low levels as during winter hibernation bouts under fluctuating conditions (both 0.03 ml O_2_^∗^g^–1*^h^–1^). In both cases torpid metabolic rates were much lower than would be expected from mere Q_10_ effects when comparing torpid $\dot{\text{V}}\,{\text{O}}_{2}$ with maximum $\dot{\text{V}}\,{\text{O}}_{2}$ at maximum *T*_skin_ (0.95–0.89 ml O_2_^∗^g^–1*^h^–1^; decrease in *T*_b_ from about 30°C to about 20°C and drop in $\dot{\text{V}}\,{\text{O}}_{2}$ to less than 5% of resting rates, instead of about 50% predicted by a Q10 of 2–3; Table 1). Thus, during the resting phases of fluctuating summer conditions, the decrease in $\dot{\text{V}}\,{\text{O}}_{2}$ was not “only” due to the suppression of thermogenesis leading to the highly labile thermoregulation tenrecs are famous for (Nicoll, 1986; Stephenson and Racey, 1994; Lovegrove and Génin, 2008; Levesque et al., 2014), but also an indication of active metabolic inhibition. Only one female, in S_const__24_, remained normothermic for longer than 24 h on two occasions. Furthermore, under the constant summer *T*_a_ (S_const__24_), elevated environmental temperatures precluded the attainment of the lowest levels of torpid $\dot{\text{V}}\,{\text{O}}_{2}$, however, $\dot{\text{V}}\,{\text{O}}_{2}$ was still lower than would be expected by Q_10_ effects alone (from 1.38 to 0.14 ml O_2_^∗^g^–1*^h^–1^; about 10% of RMR). However, at least during summer, the tenrecs needed temperature fluctuations, which included lower temperatures, to take advantage of the lowest, most energy saving levels of torpid $\dot{\text{V}}\,{\text{O}}_{2}$. In general, fluctuating temperature conditions proved to be energetically more efficient for *E. telfairi*. As temperatures are predicted to continue to increase with global climate change, this could pose a significant increase in energetic costs during torpor (Lovegrove et al., 2014a).

Despite often occurring at higher *T*_a_ (but not always, see Nowack et al., 2020), tropical hibernation and daily torpor has the potential to realize significant energy savings as well as its beneficial effect on water usage (Cooper et al., 2005; Withers et al., 2012). *E. telfairi* in our study reached minimal levels of metabolism during torpor episodes comparable to that of temperate and arctic species (0.03 ml O_2_^∗^g^–1*^h^–1^, Heldmaier et al., 2004). However, as active metabolic rates of tenrecs are lower, the relative energy savings are less pronounced. Apparently, this level of torpid metabolism seems to be at a threshold for mammalian minimal MR (see Frappell and Butler, 2004). The average torpid $\dot{\text{V}}\,{\text{O}}_{2}$ of *E. telfairi* was 0.06 ml O_2_^∗^g^–1*^h^–1^, the same as in the Malagasy lemur *C. medius* (Dausmann et al., 2009), and the greater hedgehog tenrec (0.08–0.1 O_2_^∗^g^–1*^h^–1^, Levesque and Lovegrove, 2014), indicating a general, or at least Malagasy, level for tropical hibernation. When *E. telfairi* were hibernating under the fluctuating *T*_a_ regime, $\dot{\text{V}}\,{\text{O}}_{2}$ correlated with *T*_a_ and was lower during the colder night phase compared to the warmer day phase. Together with the longer duration of hibernation bouts at lower *T*_a_ and thus fewer, energetically costly arousals, it is likely that it is energetically favorable for *E. telfairi* to hibernate at lower *T*_a_, at least down to a certain limit (see below).

In general, tenrec species have been reported to be highly heterothermic, except when they are pregnant or lactating (Stephenson and Racey, 1993a, b; Poppitt et al., 1994; Levesque and Lovegrove, 2014; Levesque et al., 2014). In our study individuals were not reproducing and became or continued to be torpid every day throughout the study. Flexible thermoregulation reduces general energy expenditure, however, also limits the ambient temperature breadth, over which an organism can function (Treat et al., 2018). Torpor, on the other hand, might counterbalance this disadvantage, broadening the temperature niche. Indeed, torpor use has been found to lower the risk of extinction in highly variable and quickly changing environments (Geiser and Turbill, 2009; Liow et al., 2009). Maximal *T*_skin_ during activity and arousal phases was similar in the two summer treatments and the two warmer winter treatments, and was in the same range as body temperature reported for intraperitoneally implanted temperature loggers by Lovegrove and Génin (2008), emphasizing the validity of our *T*_skin_ measurements. Maximal *T*_skin_ was lowest during the two arousals in the W_const__12_ conditions, suggesting limitations of the endogenous heating capacities at constantly low temperatures. In addition, there were also some “unsuccessful” arousals during W_const__12_ conditions, suggesting a lower limit for active arousal at around this temperature. Indeed, there seems to be a lower thermal limit for hibernation in *E. telfairi*. Support for this comes from Scholl (1974), who noted that *E. telfairi* was not able to arouse successfully at a *T*_a_ of 11°C. This sets an ultimate lower limit of *T*_a_ for long-term survival in this species and restricts its potential habitats. Although temperatures do drop below 10°C within the range of *E. telfairi* during winter nights, this does not occur very frequently and will be buffered even in hibernacula with low insulation capacity. More importantly, even on those coldest days, *T*_a_ will usually increase above 20°C during the day (Dausmann and Blanco, 2016), ensuring passive rewarming (contrary to Scholl, 1974).

It has been proposed that high costs of rewarming from torpor could limit the efficient employment of heterothermy, reducing energy savings achieved during torpor episodes (Wang, 1979; Humphries et al., 2003), especially for short torpor bouts. However, most terrestrial animals do not live in constant environments, but experience daily and seasonal fluctuations in *T*_a_ (Dillon and Woods, 2016; Dillon et al., 2016). Depending on the type and the insulation properties of their resting sites, environmental temperature fluctuations also translate into variable temperatures within the resting sites (Dausmann et al., 2004; Turner, 2020). These fluctuations in *T*_a_ can be used for assisted warming from torpor, especially in species living in tropical areas, where daily maximal temperatures tend to be higher than potential torpid *T*_b_ set-points, particularly during the winter seasons. Exogenous, mainly passive heating is known from several tropical heterotherms (e.g., *Microcebus murinus*, Ortmann et al., 1997; *Sminthopsis macroura*, Lovegrove et al., 1999; *Elephantulus myurus*, Mzilikazi et al., 2002; Geiser and Drury, 2003; *C. medius*, Dausmann et al., 2009; Thompson et al., 2015), and makes rewarming comparatively inexpensive with 60–85% reductions when compared to active warming (Lovegrove et al., 1999; Schmid et al., 2000; Geiser and Drury, 2003; Warnecke et al., 2008). The benefit from passive heating, either by daily fluctuations of *T*_a_ or by radiant heat, might lead to laboratory studies underestimating the energetic advantage of torpor in free-ranging mammals (Mzilikazi et al., 2002), might explain why daily torpor is common in sunny regions and might occur more frequently at low latitudes than hitherto believed (Geiser and Drury, 2003).

Previous laboratory studies with *E. telfairi* have used constant *T*_a_ for their experiments (Scholl, 1974; Poppitt et al., 1994; Künzle, 1998; Künzle et al., 2007; Oelkrug et al., 2013). In our study, fluctuating *T*_a_ treatments mimicked natural conditions. Under these conditions, *E. telfairi* also used daily *T*_a_ fluctuations for passively warming from daily torpor in summer as well as during arousals between hibernation bouts in winter. In both seasons, $\dot{\text{V}}\,{\text{O}}_{2}$ during rewarming from torpor under the intermediate, constant conditions was about double that of fluctuating temperature indicating an energetic advantage of passive heating. Under fluctuating conditions, the animals only activated endogenous heat production and became active after *T*_skin_ reached the high daytime *T*_n_ passively around noon, as evident by the sharp rise in MR above this threshold. *T*_a_ cycles lead to more uniform and synchronized *T*_skin_ patterns. In this way, *T*_a_ cycles not only help rewarming, but also synchronize the activity phases and torpor bouts of the animals, in contrast to constant *T*_a_ conditions where there was considerable variation in timing. In addition, arousals started about 6 h later under constant conditions, shortly after the lights had gone off. Possibly, for animals under constant temperature conditions light, or rather darkness, was taken as a (less stringent) cue to initiate exogenous heating.

The timing of torpor is not only important for its effective use (Körtner and Geiser, 2000), but synchronization of activity patterns could also be essential for social interactions, successful foraging bouts, and other important activities. Additional external stimuli, such as photoperiod, also affect the timing of torpor (Heldmaier et al., 1982, 1989; Aujard et al., 1998), but in the field these are often coupled to *T*_a_. Furthermore, some species (e.g., the small marsupial dunnarts, *Sminthopsis* sp.) have been shown to be insensitive to photoperiodic cues (Holloway and Geiser, 1996). A study on *E. telfairi* in an enclosure in Madagascar with natural climatic parameters by Lovegrove and Génin (2008) also found that the tenrecs used passive exogenous heating by *T*_a_ before initiating active heat production. Interestingly, they only found daily and prolonged torpor (maximum of 4 days), but not longer hibernation bouts, possibly due to the timing of the study (beginning of winter), constant supply of food, or lack of suitable hibernacula (tree hollows) in the enclosure. In our study, *E. telfairi* hibernated for several months in constant, as well as in fluctuating *T*_a_ during the hibernation season (winter). Our results show that *T*_a_ cycles can be an effective *zeitgeber* for activity and thermoregulatory rhythms, even during hibernation, and that careful consideration should be given to the choice of temperature regime under laboratory conditions.

During deep hibernation, arctic and temperate species typically display hibernation bouts of about or exceeding two weeks in length (Heldmaier et al., 2004), possibly because of the usually constant, low temperatures that arctic animals are exposed to in their hibernacula (Arnold et al., 1991; Buck and Barnes, 1999). Hibernation bout length is more flexible in tropical hibernators. Some species can exhibit very brief hibernation bouts (Reher et al., 2018), whereas mouse and dwarf lemurs can hibernate for several months without arousals, if they use poorly insulated hibernacula and *T*_b_ fluctuates passively with *T*_a_ above 30°C at least every couple of days, thus forgoing the need for active arousals with endogenous heating (Dausmann, 2014). Interestingly, fluctuating *T*_a_ triggered more frequent arousals during hibernation in *E. telfairi* in our study, therefore reducing average hibernation bout length from over eight (W_const__12_) and four (W_const__18_), to about 3.5 days. It is possible that the maximum T_skin_ that could be attained passively during the fluctuating *T*_a_ treatment was not high enough to satisfy physiological demands and thus to be settled during active arousals. This suggests a threshold temperature, below which hibernators have to actively terminate hibernation bouts after a certain time and which may vary between species, individually as well as temporally (Körtner and Geiser, 2000; Dausmann et al., 2005; Turbill et al., 2008; Lovegrove et al., 2014b). An increase in length of hibernation bouts with decreasing *T*_a_ has also been found in temperate and arctic species, e.g., the golden-mantled ground squirrel *Callospermophilus saturatus* (Geiser and Kenagy, 1988), suggesting that this temperature-dependence in hibernation bout length is universal in tropical as well as temperate and arctic hibernators. We found hibernation bout lengths comparable or even above those described by Scholl (1974) for *E. telfairi*, but contrary to the study by Lovegrove and Génin (2008), hibernation was maintained for several months. Interestingly, the only *T*_b_ available from a free-ranging tenrec (*Setifer setosus*) hibernating in variable ambient temperatures showed no evidence of periodic arousals, although in that study tree hole temperature did not drop below 18°C (Levesque et al., 2014).

As environmental temperature variations are the norm, rather than the exception (Dillon et al., 2016), this highlights the importance of incorporating temperature variability in laboratory evaluations of animal thermoregulation. The importance of incorporating realistic temperature variability in laboratory physiology has been receiving increasing attention as we seek to predict the effects of increasingly varying climates on animal survival (Vasseur et al., 2014; Dillon and Woods, 2016; Levesque et al., 2016). Our findings show that fluctuating *T*_a_ cycles not only affect the timing of arousals and the duration of hibernation bouts in the tropical hibernator *E. telfairi*, but also have an impact on the timing and shape of activity phase and torpor bouts during the non-hibernation season, as well as dramatically influencing energy expenditure in all seasons. Therefore, attempts to understand the energetics and thermoregulation of hibernators would be best served by the inclusion of more realistic temperature cycles to provide a true understanding of the conditions faced by heterotherms in the wild, especially under tropical conditions.

## Data Availability Statement

The datasets generated for this study are available on request to the corresponding author.

## Ethics Statement

The animal study was reviewed and approved by the Behörde für Gesundheit und Verbraucherschutz (BGV).

## Author Contributions

KD and JW conceived the idea and carried out the experiments. All authors contributed to the analyses of the data. JN performed the statistical analyses. KD, DL, and JN wrote the manuscript.

## Conflict of Interest

The authors declare that the research was conducted in the absence of any commercial or financial relationships that could be construed as a potential conflict of interest.
